# Predicting the outcome in confirmed COVID-19 patients with coronary artery disease: a key role for the first chest computed tomography

**DOI:** 10.1186/s43044-021-00148-7

**Published:** 2021-03-31

**Authors:** Negar Omidi, Masoumeh Lotfi-Tokaldany, Shapour Shirani, Mojtaba Salarifar, Hamidreza Poorhosseini, Seyyed Mojtaba Ghorashi, Afsaneh Aein

**Affiliations:** 1 Research Department, Tehran Heart Center, Tehran University of Medical Sciences, Tehran, Iran; 2 Cardiology Department, Tehran Heart Center, Tehran University of Medical Sciences, Tehran, Iran; 3 Imaging Department, Tehran Heart Center, Tehran University of Medical Sciences, Tehran, Iran; 4 Interventional Cardiology Department, Tehran Heart Center, Tehran University of Medical Sciences, North Kargar Street, Tehran, 1411713138 Iran

**Keywords:** Chest, Computed tomography, COVID-19, Score, Coronary artery disease

## Abstract

**Background:**

Serial chest computed tomography (CT) scans are used to detect coronavirus disease 2019 (COVID-19) pneumonia and monitor the disease course. This study investigates relationship between total severity score by first chest CT and the outcome of coronavirus COVID-19 patients with coronary artery disease (CAD).

**Results:**

A total of 48 patients with a history of CAD (mean age=60.83±3.06 years, 75% male) with positive real-time reverse transcription-polymerase chain reaction for COVID-19 were included. Outcome was defined as acute respiratory distress syndrome or death. The unadjusted and adjusted effects of the CT score on the outcome were reported through odds ratio (OR) with 95% confidence interval (CI). Outcome occurred in 17 (35.5%) patients (8 deaths). The CT score was directly and significantly correlated with the outcome in the univariate analysis (OR 1.38, 95% CI 1.12–1.70; *P*=0.003) and remained significant after adjustment for diabetes, hypertension, body mass index, and serum level of highly sensitive C-reactive protein (OR 1.51, 95% CI 1.11–2.05; *P*=0.009). Outcome rate was 24.1% in patients with a CT score <2.5, whereas it was 8.3% in patients with a CT score>2.5.

**Conclusions:**

The first chest CT score could be a robust predictor of adverse events in confirmed COVID-19 patients with coronary artery disease.

## Background

The outbreak of coronavirus disease 19 (COVID-19) originated in China in December 2019 and has continued ever since. This insidious disease is caused by a beta coronavirus named “severe acute respiratory syndrome coronavirus-2” [1].

Patients with cardiovascular disease are at risk of developing more severe and fatal forms of COVID-19. The diagnosis of COVID-19 according to the World Health Organization interim guidance is based on clinical symptoms with either positive real-time reverse transcription-polymerase chain reaction (rRT-PCR) or chest computed tomography (CT) [2]. Nonetheless, rRT-PCR is a specific marker, and the determination of the correlation between chest CT findings and rRT-PCR results poses a challenge in that it could differ according to the sensitivity of rRT-PCR and the duration from symptom initiation.

Chung et al. [3] introduced a CT quantification severity score to measure the severity of lung involvement, with the score being generated based on inflammatory lung lesions ranging from 0 to 25. Most recent studies on the COVID-19 pandemic have focused on descriptions of chest CT findings such as characteristic changes during follow-ups in serial chest CT scans and the association between CT findings and patients’ baseline characteristics [3–6].

The aforementioned findings encouraged us to focus on the role of the first chest CT findings in predicting the outcome of rRT-PCR–confirmed COVID-19 patients with history of CAD. To that end, we assessed the significance of the total severity score calculated on the basis of the first chest CT findings.

## Methods

Between 20 February and 20 April 2020, from patients who were admitted in our center [7], tertiary center for the treatment of heart diseases, with first impression of unstable angina, 106 patients were diagnosed as having COVID-19 infection. From these, 48 patients with history of CAD who did not require invasive cardiac procedures but had positive rRT-PCR for COVID-19 were enrolled. All included patients also underwent chest CT in the same hospitalization, and we retrospectively reviewed results of their chest CT. The patients’ demographic and clinical characteristics, laboratory findings, and total severity of lung involvement based on the first chest CT were recorded. The impact of the CT severity score on the in-hospital outcome was evaluated. The in-hospital endpoint, defined as a worse outcome, was considered to be death or acute respiratory distress syndrome (ARDS) requiring mechanical ventilation.

The entire study population underwent thoracic 16-slice non-contrast CT on a SOMATOM Sensation 16 CT scanner (Siemens Healthcare, Forchheim, Germany). Scoring was done semi-quantitatively, and the severity of lung involvement was measured in 5 lobes. Each lung lobe gave a 0 to 5 score. The sum of the scores of the 5 lobes determined the total score, ranging from 0 to 25 [8, 9]. In this scoring system, 0 indicates no lung involvement, and scores of 1 to 4 denote lung involvement of equal to or less than 5%, 25%, 50%, and 75%, respectively. Additionally, in cases with a minimum lung involvement of 75%, a score of 5 is given. The reporting of image analyses was based on the consensus of trained radiologists with more than 10 years of experience [10].

Patient received antiviral treatment according to the latest available guidelines [11]. Patients were treated by lopinavir/ritonavir (KALETRA) or oseltamivir with or without hydroxychloroquine.

### Statistical analysis

The categorical variables are expressed as frequencies with percentages. The normality of the distribution of the continuous variables was assessed using descriptive measures as well as their histograms. The normally distributed variables were described as the mean and the standard deviation (SD). The skewed or non-normally distributed variables were expressed as the median with 25th and 75th percentiles (interquartile range boundaries). Limitations such as the small sample size, non-normally distributed values, and wide confidence intervals (CIs) made it difficult to judge the relationship between the different variables and the outcome. Therefore, the relationship between each variable of interest and the outcome was determined through the calculation of the effect size for each variable. The standardized mean difference between the 2 study groups (with positive and negative outcomes) was calculated using the Cohen *d*, the rank-biserial correlation, and the Cohen *h* methods for the continuous (with normal and non-normal distributions) and the categorical variables, respectively. The calculated effect sizes were described as small (0.20), medium (0.50), and large (0.80) [12]. Variables with an effect size of greater than 0.20 were considered potential confounders for the relationship between the CT score and the outcome. A multivariable logistic regression model adjusted for diabetes mellitus, hypertension, the body mass index (BMI), a serum blood urea nitrogen/creatinine (BUN/Cr) ratio of greater than 20 (pre-renal azotemia), and highly sensitive C-reactive protein (hs-CRP) was established to examine the relationship between the CT score (derived from the first chest CT) and the in-hospital outcome. The unadjusted and adjusted effects of the CT score on the outcome were reported through odds ratios (ORs) with 95% CIs. The receiver operating characteristic (ROC) curve was used to test the ability of the CT score to differentiate patients with high and low risks for the worse outcome. Consistency of visual quantification between two observers was tested using intraclass correlation coefficient (ICC). ICC values were categorized as poor (<0.40), moderate (0.41–0.75), and good (>0.75). For the statistical analyses, IBM SPSS Statistics for Windows, version 25, was used.

## Results

The mean age of the participants was 60.83 ± 13.06 years (range = 25–95 years). Men accounted for 75% (*n* =36) of the study population. The most prevalent presenting symptom was chest pain in 24 (50%) patients, followed by dyspnea in 23 (47.9%). The other presenting symptoms were fever (25%), dry cough (25%), fatigue (10.4%), and muscle pain (8.3%). Table 1 summarizes the demographic and clinical characteristics of the patients on admission. With regard to antiviral treatment, 37 patients (77.1%) received hydroxychloroquine, 22 (45.8%) received KALETRA, and 13 (27.1%) received oseltamivir. In total, 4 (8.3%) patients did not receive antiviral therapy.

**Table 1:** Demographic and clinical findings of the study patients

	Total (***n***=48)
Age, mean	60.8±13.2
Male	36 (75)
Underlying cardiac disease	
History of CABG	24
History of PCI	12
History of positive coronary angiography/CT angiography	12
Three vessel disease	1
Two vessel disease	1
Mild CAD^*^	10
Concomitant valvular heart disease	5
**Symptoms**	
Chest pain	24 (50)
Dyspnea	23 (47.9)
Fever	12 (25)
Coughing	12 (25)
Muscle pain	4 (8.3)
Fatigue	5 (10.4)
**Symptom onset to chest CT**	3 days
Percentile 25th, 75th	1, 4 days
Range	0–9 days
**Risk factors and comorbidities**	
Diabetes mellitus	22 (45.8)
Hypertension	32 (66.7)
cigarette smoking	16 (33.3)
Dyslipidemia	23 (47.9)
Opium addiction	8 (16.7)
COPD	1 (2.1)
Renal failure	5 (10.4)
LVEF ≤35%	11 (22.9)
**Drug history**	
Insulin	6 (12.8)
Oral DM drugs	7 (14.9)
Statin	28 (60.9)
ARB/ACEI	26 (54.2)

*CT*, computed tomography; *COPD*, chronic obstructive pulmonary disease; *DM*, diabetes mellitus; *LVEF*, left ventricular ejection fraction; *ARB*, angiotensin II receptor blocker; *ACEI*, angiotensin-converting-enzyme inhibitor

*Mild coronary artery disease refers to 25–50% luminal stenosis in at least one of the major epicedial arteries

During the course of the hospital stay, mechanical ventilation due to respiratory failure was required for 17 (35.45%) patients, of whom 8 (16.7%) died. The median time interval between symptom onset and chest CT was 3 days (25% and 75% percentiles = 1 and 4; range = 0–9). Despite having positive rRT-PCR tests, 10 patients had normal chest CT. Results of analysis for the consistency of visual CT quantification for two independent observers showed good reproducibility with ICC = 0.988 (95% CI, 0.953–0.997).

The patients were divided into 2 groups: positive-outcome patients (*n* =17) and negative-outcome patients (*n* =31). Table 2 presents comparisons between the 2 groups. The proportion of the worse outcome was high in patients with hypertension and a BUN/Cr ratio of greater than 20 by comparison with those without hypertension and a BUN/Cr ratio of higher than 20; still, the effect size for both was weak (0.345 and 0.305, respectively). Patients with diabetes mellitus had a lower chance of having the worse outcome than those without the disease, with a weak effect size of 0.317.

**Table 2:** Univariate relationships between the demographic and clinical characteristics and the outcome

	Outcome + ***n***=17	Outcome − ***n***=31	OR (95% CI)	Effect size**
**Sex**			1.13 (0.29–4.49)	0.059
Male	13 (36.1)	23 (63.9)		
Female	4 (33.3)	8 (66.7)		
**Diabetes mellitus**			0.51 (0.15–1.730)	0.317
Yes	6 (27.3)	16 (72.7)		
No	11 (42.3)	15 (57.7)		
**Hypertension**			3.37 (0.80–4.18)	0.345
Yes	14 (43.6)	13 (56.4)		
No	3 (18.8)	13 (81.2)		
**ACEI/ARB**			0.93 (0.28–3.03)	0.073
Yes	9 (34.6)	17 (65.4)		
No	8 (38.1)	13 (61.9)		
**History of renal failure**			1.24 (0.19–8.29)	0.105
Yes	2 (40)	3 (60)		
No	15 34.9)	28 (65.1)		
**LVEF,** number of patients/total patients			0.71 (0.15–3.50)	0.158
> 35%	9/26 (34.6)	17/26 (65.4)		
≤ 35%	3/11 (27.3)	8/11 (72.7)		
LVEF*, *n*=37	52 (29, 55)	43 (32, 55)	1.01 (90.96–1.07)	0.110
**BUN/Cr > 20**			1.87 (90.51–6.88)	0.305
Yes	6 (46.2)	7 (53.8)		
No	11 (31.4)	24 (68.6)		
Age, year	61.9 ± 16.2	60.3 ± 11.2	1.01 (0.96–1.06)	0.122
BMI, *n*=44	28.8 ± 5.7	27.4 ± 4.5	1.06 (0.93–1.21)	0.284
hs-CRP*	7.4 (2.8, 12.2)	3.2 (0.7, 8.7)	1.07 (0.97–1.02)	0.327
CT score*	6 (2.5, 11)	1 (0, 2)	1.38 (1.12–1.70)	0.634
LVEF*, *n*=37	52 (29, 55)	43 (32, 55)	1.01 (90.96–1.07)	0.110

*ACEI*, angiotensin-converting enzyme inhibitor; *ARB*, angiotensin II receptor blocker; *LVEF*, left ventricular ejection fraction; *BUN*, blood urea nitrogen; *Cr*, creatinine; *CT*, computed tomography; *BMI*, body mass index; *hs-CRP*, highly sensitive C-reactive protein

*Median (25%, 75%)

**Effect size was calculated as the Cohen *h* for the categorical variables, the Cohen *d* for age and BMI, and the rank-biserial correlation for hs-CRP, the CT score, and LVEF

Patients with the positive outcome had a higher mean BMI (28.8 ± 5.7 vs 27.4 ± 4.5; effect size = 0.284), median hs-CRP (7.4 [2.8, 12.2] vs. 3.2 [0.7, 8.7]; effect size = 0.327), and median CT score (6 [2.5, 11] vs 1 [0, 2]; effect size = 0.634). While the effect size was weak for BMI and hs-CRP, the CT score had a medium effect size, indicating that a 1-score increase in the CT score was accompanied by a 38% increase in the chance of having the worse in-hospital outcome (*P* = 0.003).

To investigate whether the CT score had an independent correlation with the worse outcome, we adjusted this relationship for the confounding factors namely diabetes mellitus, hypertension, pre-renal azotemia, BMI, and hs-CRP using the multiple logistic regression analysis. After the adjustments (Table 3), the effect of the CT score on the worse outcome remained significant, and the related OR increased from 1.38 to 1.51.

**Table 3:** Adjusted and unadjusted relationships between the CT score and the outcome

CT score	OR (95% CI)	***P*** value
Unadjusted	1.38 (1.12–1.70)	0.003
Adjusted^*^	1.51 (1.11–2.49)	0.009

*Adjusted for diabetes mellitus, hypertension, body mass index, BUN/Cr>20, highly sensitive C-reactive protein

*CT*, computed tomography; *BUN*, blood urea nitrogen; *Cr*, creatinine

**Fig. 1:**
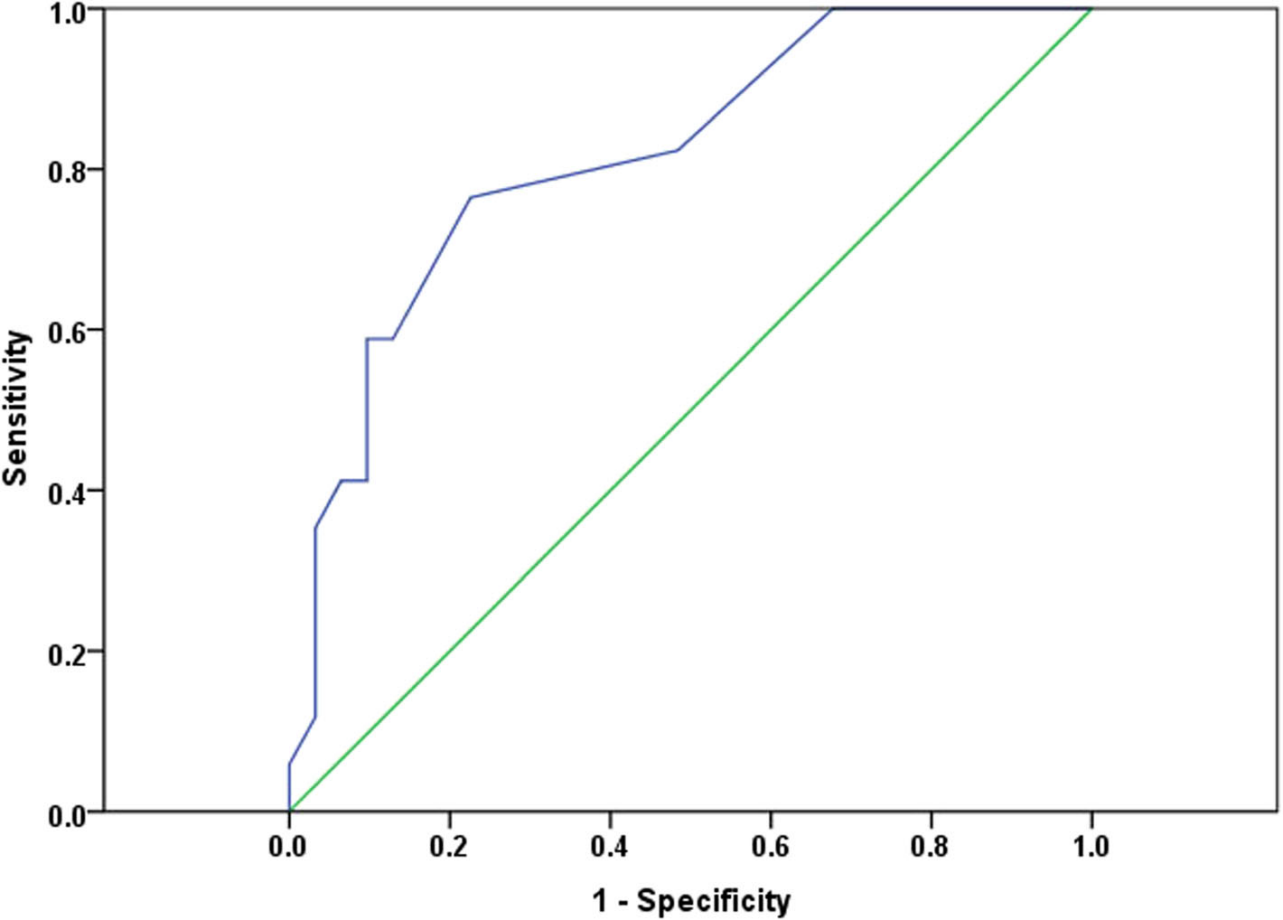
The receiver operating characteristic curve for the ability of the score from the first chest CT to discriminate patients at high risk for mechanical ventilation/death. Area under the curve 0.817 (95% CI 0.692 to 0.942)

According to the ROC analysis (Fig. 1), the area under the curve for the ability of the score from the first chest CT to distinguish patients at high risk for mechanical ventilation/death was 0.817 (95% CI 0.692 to 0.942). A cutoff of 2.5 for the first chest CT score had an accuracy of 77%, a sensitivity of 76.5% (95% CI: 52.7 to 90.5), and a specificity of 77.4% (95% CI: 60.2 to 88.6). Positive and negative predictive values for this cutoff were 65% (95% CI 43.3 to 81.9) and 85.7% (95% CI 68.5 to 94.3), respectively. Overall, 20 (41.7%) patients had a minimum CT score of 2.5. Patients with a minimum CT score of 2.5 had a worse outcome rate of 24.1% (13/20), whereas the worse outcome occurred in 4 (8.3%) of the 28 patients with a CT score of less than 2.5.

## Discussion

The present study highlights the prognostic role of the first chest CT during hospitalization in patients with COVID-19. The CT quantitative score has a vital role in predicting the outcome of those with confirmed COVID-19. This prognostic role of the first chest CT appears to be stronger than that of age, sex, BMI, hs-CRP, hypertension, and pre-renal azotemia. The discriminatory feature of our patients was that they all had a history of CAD. The study endpoint, a composite of death and ARDS requiring mechanical ventilation, occurred in 17 (35.45%) patients. The relationship between the CT score and the outcome had a large effect size (0.634) compared with that for BMI (0.284), hs-CRP (0.327), and hypertension (0.245), suggesting that the score of the first chest CT had a strong correlation with the worse outcome in comparison with the other factors.

For the diagnosis of COVID-19, Ai et al. [13] reported positive rates of 59% for rRT-PCR and 88% for chest CT imaging, with chest CT having a sensitivity of 97%. The authors also reported that 60 to 93% of their patients had initial lung involvement in chest CT even before the initial positive rRT-PCR results, although 75% of their patients with negative rRT-PCR tests had positive chest CT findings. Ai and colleagues considered 48% of these patients to be highly likely cases and 33% probable cases [13]. Consequently, chest CT has a high sensitivity for the diagnosis of COVID-19. It has also been suggested that chest CT can be regarded as a primary modality for COVID-19 detection, especially in epidemic areas [14].

Pan et al. [10] reported their chest CT scores in 4 stages: 2 ± 2 (0–6) in the early stage (0–4 days), 6 ± 4 in the progressive stage (5–8 days), 7 ± 4 in the peak stage (9–13 days), and 6 ± 4 in the absorption stage (≥ 14 days after the onset of symptoms), which is similar to our findings given the time interval from symptoms. Lung involvement is more severe 6 to 11 days (approximately on day 10) after the initiation of symptoms [6, 13]

Li et al. [8] concluded that a CT severity score with a cutoff of 7.5 was able to diagnose severe pneumonia with 100% specificity and 82.6% sensitivity. In the present study, our data showed a cutoff of 2.5 with a sensitivity of 76.5% and a specificity of 77.4%. Our literature review demonstrated that the sensitivity of chest CT for the diagnosis of COVID-19 ranges from 44 to 98% [6, 13–16], which may be because of various time durations from symptom onset to the first chest CT. For instance, the results of a study by Wang et al. [6] indicated that the sensitivity of chest CT for COVID-19 increased over time after symptom onset to 84% (95% CI 73 to 92%) and 99% (95% CI 93 to 100%) for disease days 0 to 5 and 6 to 11, respectively. In our research, the median time interval between the presentation of symptoms and chest CT was 3 days (range = 0–9 days), which may be due to the fact that most patients present to medical centers with a delay of several days following the prodromal symptoms of COVID-19. Even though in our study, the first CT scan was performed in the early days after the onset of symptoms (75% before day 5 of the disease), the results indicated the significant independent value of the first CT score in predicting in-hospital outcomes. It appears that the first CT can be considered an independent predictive factor for death and mechanical ventilation in patients with concomitant CAD and COVID-19 infection.

The risk factors of a poor prognosis in patients with COVID-19 include old age (≥ 50 years); male sex; smoking; chronic kidney disease; chronic obstructive pulmonary disease; cerebrovascular disease; elevated levels of lactate dehydrogenase, CRP, and D-dimer; decreased blood platelet and lymphocyte counts; cardiovascular disease; hypertension; and diabetes mellitus [17]. Having at least one comorbidity such as hypertension, diabetes mellitus, cardiovascular disease, and lung disease is a predictive risk factor of progression to severe disease [18]. Nevertheless, in the current study, patients with diabetes mellitus had a lower chance of having the worse outcome than those without the disease (effect size = 0.317). This observation is in line with the recent findings showing that mechanisms employed by anti-diabetic drugs such as metformin and gliptins may be protective against severe infection. It has been hypothesized that target receptors of medications commonly used to treat DM may be involved in the viral entry mechanism of SARS-CoV-2 [19]. However, further studies are required to confirm these observations.

Furthermore, elevated serum levels of CRP, erythrocyte sedimentation rates, and lactate dehydrogenase, as well as high fever, are associated with the severity of lung involvement on initial CT [4]. Obesity is also deemed an independent risk and prognostic factor for the disease severity and the need for advanced medical care in COVID-19 [20]. It can, therefore, be concluded that the value of the severity of lung involvement on admission chest CT in the context of cardiovascular disease is more robust than that of obesity. The first chest CT during workup for COVID-19 should be accorded more significance.

### Limitations

The major limitation of the present study is its small sample size, which mostly resulted in wide SDs or wide 95% CIs and precluded us from showing statistically significant differences. To overcome this limitation and evaluate the strength of each statistical claim, we calculated effect sizes to make the correlation between each variable and the outcome comparable with one another. However, a future longitudinal study with follow-up CT results would be of value.

## Conclusions

The CT score based on the first chest CT findings could be used as a practical predictor of the outcome in rRT-PCR–positive COVID-19 patients with a history of CAD. The significance of the CT severity score in the prediction of the outcome in patients with coronary artery disease may be comparable with that of comorbidities such as hypertension.

## Data Availability

The datasets analyzed during the current study are not publicly available due to the institutional policy but are available from the corresponding author on reasonable request.
